# On the Resistance to Relapse to Cocaine-Seeking Following Impairment of Instrumental Cocaine Memory Reconsolidation

**DOI:** 10.3389/fnbeh.2019.00242

**Published:** 2019-10-15

**Authors:** Marc T. J. Exton-McGuinness, Mohamed L. Drame, Charlotte R. Flavell, Jonathan L. C. Lee

**Affiliations:** School of Psychology, University of Birmingham, Birmingham, United Kingdom

**Keywords:** cocaine, addiction, instrumental, memory, reconsolidation, relapse

## Abstract

Reconsolidation normally functions to update and maintain memories in the long-term. However, this process can be disrupted pharmacologically to weaken memories. Exploiting such experimental amnesia to disrupt the maladaptive reward memories underpinning addiction may provide a novel therapeutic avenue to prevent relapse. Here, we tested whether targeted disruption of the reconsolidation of instrumental (operant) lever pressing for cocaine resulted in protection against different forms of relapse in a rat self-administration model. We first confirmed that systemic injection of the non-competitive N-methyl–D-aspartate receptor (NMDAR) antagonist MK-801 did impair reconsolidation to reduce spontaneous instrumental drug-seeking memory at test. This deficit was not rescued by pharmacological induction of stress with the anxiogenic α_2_-noradrenergic receptor antagonist yohimbine. In contrast, cocaine-seeking was restored to control levels following priming with cocaine itself, or presentation of a cocaine-associated cue. These results suggest that while stress-induced relapse can be reduced by disruption of instrumental memory reconsolidation, the apparent sparing of the pavlovian cue-drug memory permitted other routes to relapse. Therefore, future reconsolidation-based therapeutic strategies for addictive drug-seeking may need to target both instrumental and pavlovian memories.

## Introduction

Memories are constantly evolving through a constructive process that serves to update their content. One core mechanism of memory updating is believed to be memory reconsolidation (Nader, 2003; Lee, 2009). Following appropriate retrieval, believed to involve some form of prediction error (Exton-McGuinness et al., 2015), a memory can be destabilized, requiring that it is subsequently restabilized in a reconsolidation process that necessitates activity at N-methyl–D-aspartate receptors (NMDARs), gene expression, and protein synthesis (Tronson and Taylor, 2007). Pharmacologically impairing the restabilization of a memory during the reconsolidation phase can induce amnesia. This finding has generated much interest in treating disorders underpinned by maladaptive memories (Kindt et al., 2009; Taylor et al., 2009; Milton, 2013).

It has been suggested that addictions are driven by the formation of a maladaptive “habit” memory (Milton and Everitt, 2012), which supports the underlying behavioral compulsion to seek drugs, regardless of consequences, that characterizes the state of addiction (Koob and Volkow, 2010). Importantly, both instrumental (operant) associations and pavlovian conditioned stimuli (CSs) contribute to the performance and maintenance of drug-seeking behaviors (Belin et al., 2013; Hogarth et al., 2013). This is critical in understanding relapse, as each bout of relapse is often precipitated by exposure to stress, the drug itself or a drug-associated CS (Bossert et al., 2005).

Addictive drug memory reconsolidation studies have predominantly focussed on pavlovian cue-drug memories, showing that these memories can be disrupted to reduce cue-induced drug-seeking and relapse in both animal models (Milton et al., 2008a) and human populations (Xue et al., 2012). However, given that relapse can also occur following induction of stress (Shaham et al., 2000) or re-exposure to the drug itself (Jaffe et al., 1989), and that impairment of pavlovian cocaine memory reconsolidation does not appear to protect against cocaine-induced relapse (Sanchez et al., 2010), the potential for long-lasting therapeutic benefit may be limited.

As addictive drug-seeking requires intact instrumental memory and expression, it is possible that targeting the instrumental memory for reconsolidation disruption might provide more robust protection against stress- and drug-induced relapse. We have previously demonstrated that instrumental memories can be impaired by the NMDAR antagonist MK-801, under conditions of brief training with both sucrose and intravenous cocaine reinforcement (Exton-McGuinness and Lee, 2015), and more extensive sucrose reinforcement (Exton-McGuinness et al., 2014), findings that are consistent with the effects of NMDAR antagonists to impair memory reconsolidation in other settings (Sadler et al., 2007; Winters et al., 2009; Reichelt and Lee, 2012, 2013). In the present study, we used an intravenous (i.v.) self-administration paradigm to investigate: (1) whether reconsolidation-disruption of instrumental cocaine memory can reduce spontaneous drug-seeking after an established period of self-administration; and (2) whether reconsolidation-disruption of instrumental memory could also provide resistance to relapse triggered *via* pharmacological stress [mimicking physiological and psychological stress with the anxiogenic α_2_-noradrenergic receptor antagonist yohimbine (Stine et al., 2002; Feltenstein and See, 2006)], re-exposure to cocaine, or presentation of a drug-associated cue.

## Materials and Methods

Subjects were 124 experimentally naïve male Hooded-Lister rats (Charles River, UK) weighing 250–350 g (median 275 g) at the beginning of the experiment. Rats were kept in a conventional animal facility on a 12 h light/dark cycle (lights on 0700), housed in quads in individually ventilated cages with two levels; the lower contained aspen chip bedding. Environmental enrichment was available in the form of wood chew blocks and paper house. Food and water were provided *ad libitum*. Experimental sessions took place 0800–1600 each day. At the end of the experiment, all animals were humanely killed *via* a rising concentration of CO_2_. All procedures were approved by a local ethical review board and carried out in accordance with the UK Animals (Scientific Procedures) Act 1986, Amendment Regulations 2012 (PPL P8B15DC34 and PPL P3B19B9D2).

### Surgical Procedures

Drinking water was supplemented with the broad-spectrum antibiotic Baytril for 7 days, beginning 3 days pre-operatively. Rats were anesthetized using isoflurane (5% induction, 2%–3% maintenance), and administered peri- and post-operative buprenorphine; their diet was also supplemented with the non-steroidal anti-inflammatory Carprofen for 1 days pre-operatively, and 3 days post. Rats were allowed a minimum of 7 days recovery, during which they were singly housed on Puracel bedding; rats were rehoused in quads at the start of experimental procedures.

Surgeries were carried out aseptically during which rats were implanted with chronic indwelling jugular vein catheters (Polyurethane Intravascular Tubing; Instech, PA, USA) aimed at the left vena cava, secured with silk suture, and exteriorized on the dorsum with a small plastic implant (PlasticsOne, Roanoke, VA, USA; 313-000BM-15-5UP/1/SPC) secured to the skin with a 1 inch mesh.

### Drugs

Cocaine HCl (Macfarlane Smith Ltd, UK) was dissolved in sterile saline to a concentration of 2.5 mg/ml; i.v. infusions of 0.1 ml over 5.6 s could be obtained during training and reactivation. Infusion dosage was based upon established literature (Di Ciano and Everitt, 2001). For cocaine-primed reinstatement, 10 mg/kg of cocaine solution was injected intraperitoneally (i.p.) immediately before the behavioral session; this dose is known to reinstate lever pressing for cocaine (Brimijoin et al., 2008).

MK-801 (AbCam, UK) was dissolved in sterile saline to a concentration of 0.1 mg/ml. Thirty minutes prior to memory reactivation rats were injected i.p. with 0.1 mg/kg of MK-801 or saline vehicle. This dose is established to disrupt instrumental memory reconsolidation (Exton-McGuinness et al., 2014; Exton-McGuinness and Lee, 2015). Injections were assigned systematically by cage, randomly within each cage.

Yohimbine (Sigma-Aldrich, UK) was dissolved in sterile saline to a concentration of 1.25 mg/ml. For stress-induced reinstatement, yohimbine was administered 30 min prior to testing (1.25 mg/kg). This dose is established to reinstate drug-seeking in cocaine settings (Feltenstein and See, 2006).

### Behavioral Procedures

Behavioral sessions took place in eight operant boxes (MedAssociates, VT), as described previously (Exton-McGuinness and Lee, 2015). Prior to each session catheters were flushed with heparinized saline (0.1 ml, 30 IU/ml). Catheters were then connected to an infusion pump (MedAssociates, VT, USA) and secured with a spring tether. The study consisted of five experiments each with the same training and reactivation protocols, however, testing was conducted in one of five conditions: (1) spontaneous seeking; (2) CS-induced relapse; (3) yohimbine-induced stress; (4) cocaine priming; and (5) yohimbine + CS relapse.

#### Training

Rats were trained to self-administer cocaine for 10 days, on a fixed-ratio-1 schedule. Two levers were extended into the chamber; one assigned the “active” cocaine-reinforced lever. Lever assignments were made systematically prior to the start of training. Active responses triggered delivery of a single cocaine infusion and a 20-s illumination of a light CS above the active lever, during which the houselight went out. Both levers remained extended throughout the session, and inactive responses had no consequence. A 20-s timeout was enforced between infusions. Training sessions lasted 90 min, or were terminated when a maximum of 30 infusions were received.

#### Reactivation

Forty-eight hours after the final training day, rats were injected i.p. with MK-801, or saline, 30 min prior to a variable-ratio (VR5) reactivation session (Exton-McGuinness and Lee, 2015). VR5 required a random number of active lever presses to gain an infusion (mean: 5, range: 1–9). Reactivation lasted 20 min, or until the maximum of 20 infusions was obtained. Cocaine infusions were as in training: accompanied by a CS (20 s) with a 20-s time-out between infusions.

#### Testing

The following day, responding was tested in a 90-min extinction session. Levers were extended throughout, and no cocaine was delivered. The drug-paired CS was only presented in the CS-relapse and Yohimbine+CS-relapse conditions and was absent in all other tests. For yohimbine-, yohimbine+CS and cocaine-induced relapse, rats were pre-treated with yohimbine or cocaine, respectively.

### Statistical Analyses

Data are represented as mean ± SEM, and were analyzed using JASP 0.9.1 (JASP Team, 2016). The primary analyses were frequentist, with alpha = 0.05 and $\eta_{\text{p}}^{2}$ reported as an index of effect size. Parametric assumptions were tested using Levene’s test for equality of variances. For acquisition data, Session was included as a factor; where appropriate a Greenhouse-Geisser correction was applied to correct for sphericity violations. The primary outcome of interest was the discriminated responding between the active and inactive levers. Drug treatment was assigned pseudo-randomly to produce two groups similarly performing during training. Test sessions were analyzed divided into two equal time bins (1 vs. 2) as per previous analytical approaches (Lee et al., 2006). For the Yohimbine+CS relapse experiment, the data were analyzed with MK-801 and Yohimbine drug factors in independent analyses, with planned follow-up comparisons (e.g., effect of MK-801 in Yohimbine groups). We also report BF_Inclusion_ from parallel Bayesian analyses (Cauchy prior *r* = 0.707) as an estimate of posterior probability.

Four rats were culled prior to the start of the experiment owing to problems during surgical recovery, and seven during training due to biological rejection of catheters. Eight rats were excluded due to catheter blockages, four rats were excluded due to equipment failures, and two rats were excluded owing to a failure to learn the task (<25 infusions during training). Four rats were excluded from the Yohimbine+CS relapse experiment as their test data were >2 SD from the group mean.

## Results

### Experiment 1: Disruption of Spontaneous Drug-Seeking

**Figure 1:**
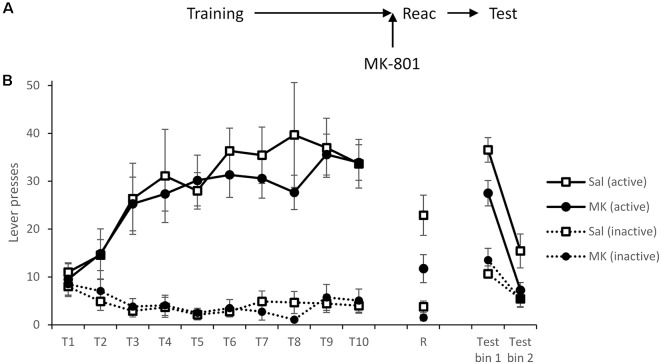
Long-term lever pressing was significantly impaired at test following administration MK-801 in conjunction with a reactivation session. **(A)** Schematic of the experiment. **(B)** Rats learned to self-administer cocaine over training days 1–10 (T1–T10). At reactivation (R), overall responding was acutely reduced by MK-801 (*n* = 12) administration compared to saline-injected controls (*n* = 9), however, this was not specific to either lever. In a 90-min extinction test, previously MK-801 treated rats made significantly fewer responses on the active (previously cocaine paired) lever. Data are presented as summary mean ± SEM.

Differences at test could not be attributed to prior differences during training (Figure 1) as both groups learned to respond predominantly on the active lever and performed similarly prior to reactivation (MK-801 × Lever × Session: *F*_(5.2,97.9)_ = 0.20, *p* = 0.97, $\eta_{\text{p}}^{2}$ = 0.010, BF_inc_ < 0.001; MK-801 × Lever: *F*_(1,19)_ = 0.27, *p* = 0.61, $\eta_{\text{p}}^{2}$ = 0.014, BF_inc_ = 0.13; MK-801: *F*_(1,19)_ = 0.33, *p* = 0.57, $\eta_{\text{p}}^{2}$ = 0.017, BF_inc_ = 0.12).

Forty-eight hours after training, rats were injected i.p. with MK-801 (or saline vehicle) 30 min prior to a short VR5 reactivation session in which the reward contingency was altered. Responding of MK-801 treated individuals was generally reduced (MK-801: *F*_(1,19)_ = 6.97, *p* = 0.016, $\eta_{\text{p}}^{2}$ = 0.27, BF_inc_ = 3.17); however, rats still showed some evidence of discrimination between the levers (MK-801 × Lever: *F*_(1,19)_ = 3.19, *p* = 0.090, $\eta_{\text{p}}^{2}$ = 0.14, BF_inc_ = 3.87).

On the following day, spontaneous cocaine-seeking performance was tested in extinction, in the absence of cocaine or the CS. MK-801-treated rats showed poorer discriminated responding on the active vs. inactive lever across the test session (MK-801 × Lever: *F*_(1,19)_ = 12.7, *p* = 0.002, $\eta_{\text{p}}^{2}$ = 0.40, BF_inc_ = 67.9; MK-801 × Lever × Bin: *F*_(1,19)_ = 0.48, *p* = 0.50, $\eta_{\text{p}}^{2}$ = 0.025, BF_inc_ = 1.87). This suggests MK-801 successfully disrupted instrumental cocaine memory reconsolidation.

### Experiment 2: CS-Induced Relapse

**Figure 2:**
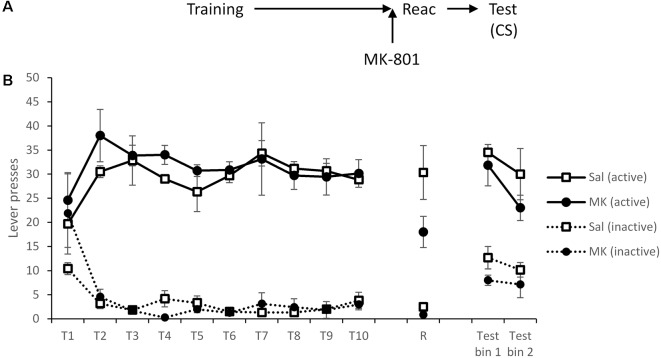
Presentation of a cocaine-paired conditioned stimulus (CS) maintained lever pressing at test, regardless of prior disruption of reconsolidation with MK-801. **(A)** Schematic of the experiment. **(B)** Rats acquired cocaine-seeking successfully over training days 1–10 (T1–T10). At reactivation (R), lever pressing was slightly reduced by MK-801 (*n* = 7) compared to saline controls (*n* = 6), although this was not statistically significant. During testing, active lever responses triggered a 1 s presentation of the cue light. This was sufficient to recover responding of MK-801 treated rats to control levels. Data are presented as summary mean ± SEM.

A second cohort of rats was trained as before with no group differences prior to reactivation and drug administration (Figure 2; MK-801 × Lever × Session: *F*_(3.3,36.8)_ = 0.44, *p* = 0.75, $\eta_{\text{p}}^{2}$ = 0.038, BF_inc_ = 0.001; MK-801 × Lever: *F*_(1,11)_ = 0.12, *p* = 0.74, $\eta_{\text{p}}^{2}$ = 0.011, BF_inc_ = 0.099; MK-801: *F*_(1,11)_ = 0.36, *p* = 0.56, $\eta_{\text{p}}^{2}$ = 0.031, BF_inc_ = 0.12).

After a 48-h rest period, rats were administered MK-801 or saline as previously prior to a VR5 reactivation. There were no significant group differences in lever responding (MK-801 × Lever: *F*_(1,11)_ = 1.46, *p* = 0.25, $\eta_{\text{p}}^{2}$ = 0.12, BF_inc_ = 1.44); however as previously observed, response rates were generally, although not significantly, lower in the MK-801 group (MK-801: *F*_(1,11)_ = 3.08, *p* = 0.11, $\eta_{\text{p}}^{2}$ = 0.22, BF_inc_ = 0.95).

At test, active responses triggered a 1-s presentation of the CS light above the lever. Under these test conditions, there was no evidence for an impairment in discriminated responding in previously MK-801-treated rats (MK-801 × Lever: *F*_(1,11)_ = 0.015, *p* = 0.90, $\eta_{\text{p}}^{2}$ = 0.001, BF_inc_ = 0.35; MK-801 × Lever × Bin: *F*_(1,11)_ = 0.71, *p* = 0.42, $\eta_{\text{p}}^{2}$ = 0.061, BF_inc_ = 0.061; MK-801: *F*_(1,11)_ = 0.65, *p* = 0.44, $\eta_{\text{p}}^{2}$ = 0.056, BF_inc_ = 0.33). Therefore, the cocaine-associated light cue was able to recover cocaine-seeking.

### Experiment 3: Yohimbine-Induced Relapse

**Figure 3:**
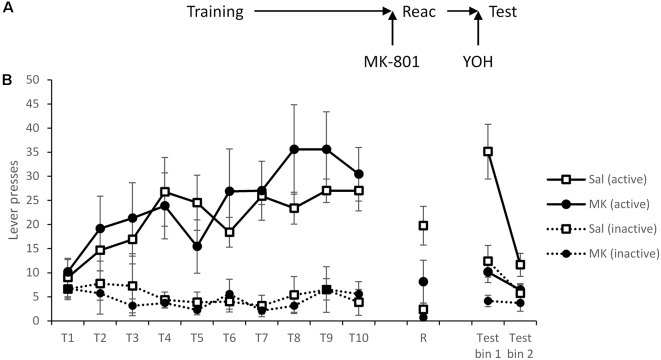
Induction of stress using the α_2_ antagonist yohimbine failed to reinstate lever pressing following reconsolidation-disruption with MK-801. **(A)** Schematic of the experiment. **(B)** Rats learned to lever press for cocaine over training days 1–10 (T1–T10). At reactivation (R), overall lever pressing was acutely reduced by MK-801 (*n* = 7) compared to saline controls (*n* = 8), however, this was not specific to either lever. Thirty minutes prior to testing all rats were injected with yohimbine and tested in extinction (with no discrete stimuli present). Rats previously treated with MK-801 remained significantly impaired, suggesting stress is not sufficient to rescue performance following disruption of instrumental memory. Data is presented as summary mean ± SEM.

A third cohort learned to self-administer cocaine, with treatment groups performing similarly prior to reactivation (Figure 3; MK-801 × Lever × Session: *F*_(9,117)_ = 1.01, *p* = 0.44, $\eta_{\text{p}}^{2}$ = 0.072, BF_inc_ = 0.009; MK-801 × Lever: *F*_(1,13)_ = 0.53, *p* = 0.48, $\eta_{\text{p}}^{2}$ = 0.039, BF_inc_ = 0.35; MK-801: *F*_(1,13)_ = 0.19, *p* = 0.67, $\eta_{\text{p}}^{2}$ = 0.014, BF_inc_ = 0.20).

At reactivation there was again evidence for an acute overall reduction in responding following MK-801 administration (MK-801: *F*_(1,13)_ = 5.91, *p* = 0.030, $\eta_{\text{p}}^{2}$ = 0.31, BF_inc_ = 1.99), and although it is somewhat unclear statistically whether this was specific to either lever (MK-801 × Lever: *F*_(1,13)_ = 2.73, *p* = 0.122, $\eta_{\text{p}}^{2}$ = 0.17, BF_inc_ = 3.1), numerically the reduction was apparent across both levers.

Thirty minutes prior to testing all rats were injected with yohimbine. Under these test conditions MK-801-treated rats had impaired discrimination (MK-801 × Lever: *F*_(1,13)_ = 7.51, *p* = 0.017, $\eta_{\text{p}}^{2}$ = 0.37, BF_inc_ = 48.2) that was time-dependent within the session (MK-801 × Lever × Bin: *F*_(1,13)_ = 4.62, *p* = 0.051, $\eta_{\text{p}}^{2}$ = 0.26, BF_inc_ = 26.7). Analysis of simple effects revealed a more obvious effect in the first half of the session (MK-801 × Lever: *F*_(1,13)_ = 6.52, *p* = 0.024, $\eta_{\text{p}}^{2}$ = 0.33, BF_inc_ = 16.7) than in the second half (MK-801 × Lever: *F*_(1,13)_ = 3.03, *p* = 0.11, $\eta_{\text{p}}^{2}$ = 0.19, BF_inc_ = 2.1; MK-801: *F*_(1,13)_ = 2.47, *p* = 0.14, $\eta_{\text{p}}^{2}$ = 0.16, BF_inc_ = 1.5). Therefore, yohimbine failed to recover the MK-801-induced deficit in instrumental cocaine-seeking.

### Experiment 4: Yohimbine+CS-Induced Relapse

**Figure 4:**
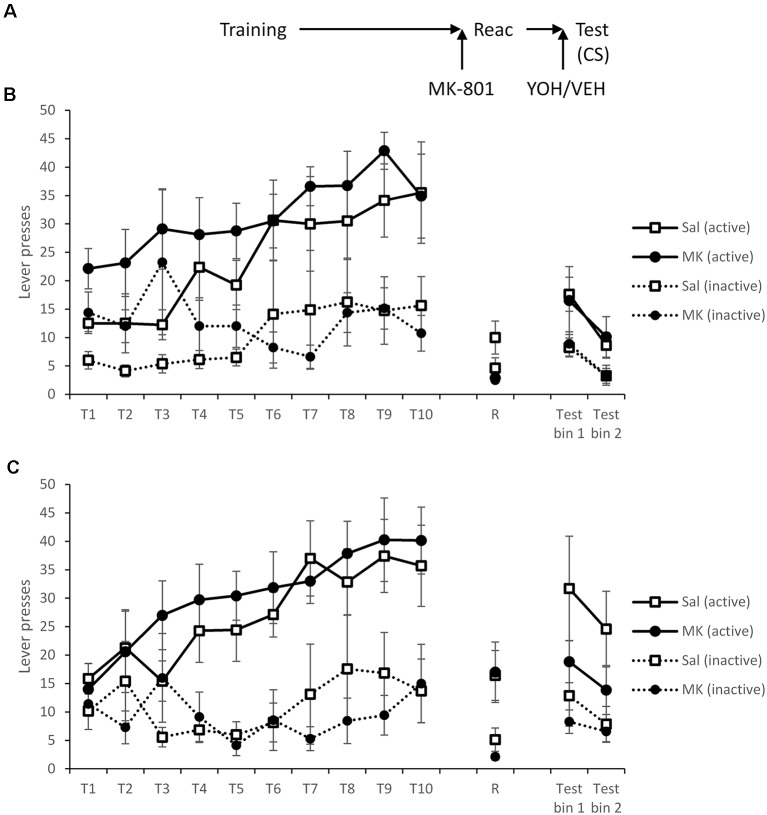
Administration of yohimbine did elevate instrumental responding in the presence of the CS, but not obviously in MK-801-treated rats. **(A)** Schematic of the experiment. **(B)** Rats receiving vehicle prior to test. **(C)** Rats receiving yohimbine prior to test. Rats learned to lever press for cocaine over training days 1–10 (T1–T10). At reactivation (R), there was no acute effect of MK-801 on performance, but the groups did differ depending on whether they subsequently received yohimbine. Thirty minutes prior to testing rats were injected with yohimbine or vehicle and were tested with active lever presses reinforced by the CS. Yohimbine elevated test responding, more obviously in saline-treated rats. CS presentation recovered responding in the absence of yohimbine, but less obviously so with pre-test yohimbine. Data are presented as summary mean ± SEM (yohimbine groups: *n* = 7; vehicle groups: *n* = 8).

Another cohort learned to self-administer cocaine as before with no significant group differences (Figure 4; MK-801 × Lever × Session: *F*_(4.4,122.7)_ = 0.66, *p* = 0.64, $\eta_{\text{p}}^{2}$ = 0.023, BF_inc_ = 0.086; MK-801 × Lever: *F*_(1,28)_ = 1.40, *p* = 0.25, $\eta_{\text{p}}^{2}$ = 0.048, BF_inc_ = 2.06; MK-801: *F*_(1,28)_ = 1.32, *p* = 0.26, $\eta_{\text{p}}^{2}$ = 0.045, BF_inc_ = 0.84; YOH × Lever × Session: *F*_(4.3,119.6)_ = 0.30, *p* = 0.89, $\eta_{\text{p}}^{2}$ = 0.011, BF_inc_ < 0.001; YOH × Lever: *F*_(1,28)_ = 0.31, *p* = 0.58, $\eta_{\text{p}}^{2}$ = 0.011, BF_inc_ = 0.096; YOH: *F*_(1,28)_ < 0.001, *p* = 0.97, $\eta_{\text{p}}^{2}$ = 0.000, BF_inc_ = 0.10).

Forty-eight hours after training rats were, as previously, injected with MK-801 or vehicle prior to reactivation. There was no significant acute effect of MK-801 treatment during this session (MK-801 × Lever: *F*_(1,28)_ = 0.076, *p* = 0.79, $\eta_{\text{p}}^{2}$ = 0.003, BF_inc_ = 0.57; MK-801: *F*_(1,28)_ = 1.76, *p* = 0.20, $\eta_{\text{p}}^{2}$ = 0.059, BF_inc_ = 0.57). In contrast, the groups that would subsequently be treated with yohimbine responded more than those to be administered saline (YOH × Lever: *F*_(1,28)_ = 10.9, *p* = 0.003, $\eta_{\text{p}}^{2}$ = 0.28, BF_inc_ = 47.9), which was surprising given the lack of any difference during training. However, this effect was only observed in MK-801-injected rats (YOH × Lever: *F*_(1,13)_ = 9.30, *p* = 0.009, $\eta_{\text{p}}^{2}$ = 0.42, BF_inc_ = 20.1), and not in the saline control condition (YOH × Lever: *F*_(1,13)_ = 2.24, *p* = 0.16, $\eta_{\text{p}}^{2}$ = 0.15, BF_inc_ = 1.30).

Rats were injected with yohimbine or vehicle 30 min prior to a test, in which active lever presses were reinforced by the light CS. In order to evaluate the capacity of yohimbine to elevate responding at test, we analyzed first the overall effect of yohimbine (Figures 4B,C). There was evidence that yohimbine did increase performance across the test (YOH × Lever: *F*_(1,28)_ = 4.46, *p* = 0.044, $\eta_{\text{p}}^{2}$ = 0.14, BF_inc_ = 3.84; YOH × Lever × Bin: *F*_(1,28)_ = 0.005, *p* = 0.95, $\eta_{\text{p}}^{2}$ = 0.000, BF_inc_ = 0.22). Planned comparisons revealed evidence for the yohimbine-induced enhancement in the non-MK-801-treated rats (YOH × Lever: *F*_(1,13)_ = 4.98, *p* = 0.044, $\eta_{\text{p}}^{2}$ = 0.28, BF_inc_ = 11.9; YOH × Lever × Bin: *F*_(1,13)_ = 0.34, *p* = 0.57, $\eta_{\text{p}}^{2}$ = 0.025, BF_inc_ = 0.53), but not in the MK-801-treated group (YOH × Lever: *F*_(1,13)_ = 0.37, *p* = 0.55, $\eta_{\text{p}}^{2}$ = 0.028, BF_inc_ = 0.33; YOH × Lever × Bin: *F*_(1,13)_ = 0.10, *p* = 0.75, $\eta_{\text{p}}^{2}$ = 0.008, BF_inc_ = 0.08; YOH: *F*_(1,13)_ = 0.38, *p* = 0.55, $\eta_{\text{p}}^{2}$ = 0.029, BF_inc_ = 0.33). Therefore, there was evidence that pre-test yohimbine was able to elevate cocaine-seeking.

There was no overall effect of MK-801 (MK-801 × Lever: *F*_(1,28)_ = 2.01, *p* = 0.17, $\eta_{\text{p}}^{2}$ = 0.067, BF_inc_ = 0.74; MK-801 × Lever × Bin: *F*_(1,28)_ = 0.082, *p* = 0.78, $\eta_{\text{p}}^{2}$ = 0.003, BF_inc_ = 0.11; MK-801: *F*_(1,28)_ = 1.01, *p* = 0.33, $\eta_{\text{p}}^{2}$ = 0.035, BF_inc_ = 0.48). Planned comparisons revealed no effect of MK-801 in the non-yohimbine groups (Figure 4B; MK-801 × Lever: *F*_(1,14)_ = 0.004, *p* = 0.95, $\eta_{\text{p}}^{2}$ = 0.000, BF_inc_ = 0.30; MK-801 × Lever × Bin: *F*_(1,14)_ = 0.24, *p* = 0.63, $\eta_{\text{p}}^{2}$ = 0.017, BF_inc_ = 0.072; MK-801: *F*_(1,14)_ = 0.009, *p* = 0.93, $\eta_{\text{p}}^{2}$ = 0.001, BF_inc_ = 0.28), with somewhat less clear evidence in the yohimbine-treated groups (Figure 4C; MK-801 × Lever: *F*_(1,12)_ = 3.26, *p* = 0.096, $\eta_{\text{p}}^{2}$ = 0.21, BF_inc_ = 0.74; MK-801 × Lever × Bin: *F*_(1,12)_ = 0.058, *p* = 0.81, $\eta_{\text{p}}^{2}$ = 0.005, BF_inc_ = 0.099; MK-801: *F*_(1,12)_ = 1.67, *p* = 0.22, $\eta_{\text{p}}^{2}$ = 0.12, BF_inc_ = 0.48). Therefore, it remains unclear whether combination of yohimbine and the CS was able to recover the lever responding fully.

### Experiment 5: Cocaine-Induced Relapse

**Figure 5:**
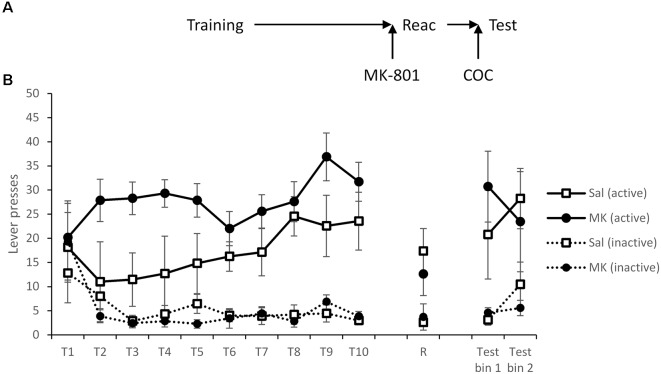
Administration of a cocaine-priming injection successfully rescued lever pressing on the active drug-lever, following MK-801 induced disruption of reconsolidation. **(A)** Schematic of the experiment. **(B)** Rats acquired cocaine-seeking successfully over the training period (T1–T10), although there were differences between the groups. At reactivation (R), overall lever pressing was slightly reduced by MK-801 administration (*n* = 7), although this was not significantly different to saline controls (*n* = 9). Immediately prior to testing rats were injected with 10 mg/kg cocaine, which subsequently rescued responding on the active lever in the MK-801 group. There was no difference at test between the groups, even when corrected for training history. Data are presented as summary mean ± SEM.

The final cohort learned to self-administer cocaine. There was evidence for an overall difference between the groups in their discriminated responding (Figure 5; MK-801 × Lever: *F*_(1,14)_ = 4.87, *p* = 0.044, $\eta_{\text{p}}^{2}$ = 0.26, BF_inc_ = 792.0; MK-801 × Lever × Session: *F*_(2.3,32.5)_ = 1.08, *p* = 0.36, $\eta_{\text{p}}^{2}$ = 0.071, BF_inc_ = 0.009), with the rats to be injected with MK-801 performing to a higher level. This is somewhat problematic for evaluating post-learning differences in behavior, and may explain the lack of acute effect of MK-801 on the VR5 reactivation session (MK-801 × Lever: *F*_(1,14)_ = 0.60, *p* = 0.45, $\eta_{\text{p}}^{2}$ = 0.041, BF_inc_ = 0.70; MK-801: *F*_(1,14)_ = 0.33, *p* = 0.57, $\eta_{\text{p}}^{2}$ = 0.023, BF_inc_ = 0.46), in that the MK-801-injected rats might have been expected to perform better than saline controls on the basis of training history.

Twenty-four hours after reactivation, rats were injected i.p. with cocaine immediately before testing commenced. Under these test conditions, there was no evidence for an impairment in discriminated responding in previously MK-801-treated rats (MK-801 × Lever: *F*_(1,14)_ = 0.022, *p* = 0.65, $\eta_{\text{p}}^{2}$ = 0.016, BF_inc_ = 0.36; MK-801 × Lever × Bin: *F*_(1,14)_ = 1.12, *p* = 0.31, $\eta_{\text{p}}^{2}$ = 0.074, BF_inc_ = 0.064; MK-801: *F*_(1,14)_ = 0.004, *p* = 0.95, $\eta_{\text{p}}^{2}$ = 0.000, BF_inc_ = 0.30). In an exploratory analysis to control partially for the different training history between the two groups, we reanalyzed the total active lever responses across the test session using an ANCOVA, with total rewards earned during training as the covariate. This analysis provided further support for there being no difference between the groups at test (*F*_(1,13)_ = 0.035, *p* = 0.85, $\eta_{\text{p}}^{2}$ = 0.003, BF_inc_ = 0.45). Therefore, cocaine priming was able to recover cocaine-seeking.

## Discussion

In the present studies, the combination of instrumental cocaine memory reactivation in a brief VR5 session and pre-reactivation systemic treatment with MK-801 reduced subsequent cocaine-seeking under certain test conditions. Reduced cocaine-seeking was observed in a test of spontaneous drug-seeking, as well as following pharmacological challenge with yohimbine. However, apparently normal seeking behavior was observed in tests of cue-induced and cocaine-induced cocaine-seeking. Therefore, while the targeting of instrumental cocaine memory reconsolidation has complementary beneficial effects to disruption of pavlovian cocaine memory reconsolidation, both appear not to confer resistance to cocaine-induced relapse.

Administration of the NMDAR antagonist MK-801 prior to VR5 reactivation reduced long-term responding for cocaine on the next day when tested in the absence of explicit precipitants of relapse. This is consistent with our previous observations that memory reactivation involving a shift to a variable reward contingency successfully destabilizes instrumental sucrose memory, allowing reconsolidation to be disrupted with MK-801 (Exton-McGuinness et al., 2014). A similar finding was also observed for weakly-trained instrumental memories for both sucrose and cocaine reward (Exton-McGuinness and Lee, 2015). Here, we implemented an adapted protocol in which our VR5 reactivation was given 48 h after training, as delaying the reactivation session appears to facilitate destabilization in sucrose settings (Cheng C., Lee J.L.C. and Exton-McGuinness M.T.J., unpublished observations). Given our previous demonstration that the decrease in lever pressing is due to disruption of instrumental memory (Exton-McGuinness and Lee, 2015), it is highly likely that the present reduction in cocaine-seeking is similarly caused by destabilization and disruption of instrumental cocaine memory. That said, the seemingly consistent effect of pre-reactivation MK-801 to lower responding during the reactivation session is in marked contrast to the elevation of responding observed in our previous instrumental sucrose memory reconsolidation study (Exton-McGuinness et al., 2014) and might point to alternative interpretations. If MK-801 were acutely affecting memory retrieval, there remains the possibility that such an impairment may be long-lasting. Alternatively, the effect of MK-801 to increase locomotor activity (Hargreaves and Cain, 1995) could become linked to memory retrieval, thereby impacting upon later test performance. This latter suggestion bears some similarity to the recent hypothesis that reconsolidation impairments can be interpreted not as mnemonic disruptions, but as the integration of new information that influences subsequent memory retrieval (Gisquet-Verrier and Riccio, 2018).

Importantly for future clinical application, reconsolidation-disruption of lever pressing was not recovered by the anxiogenic α_2_ antagonist yohimbine. Yohimbine has been observed to reinstate extinguished lever pressing, for cocaine (Feltenstein and See, 2006), nicotine (Feltenstein et al., 2012) and heroin (Banna et al., 2010) at the doses used here. However, in the present study, no recovery of responding was observed in previously MK-801-treated animals. This result indicates an inability of (at least pharmacological) stress to rescue the reconsolidation deficit, even though the same stress induction manipulation was able to elevate test responding in a control condition, compared to vehicle pre-treatment (Yohimbine+CS experiment).

In contrast to the effects of yohimbine, response-contingent CS presentation rescued performance to control levels. This demonstrates firstly that any deficits in lever pressing caused by MK-801 administration were not due to any motor incapacity to respond, as cocaine-seeking was recovered to apparently normal levels under certain test conditions. Secondly, pavlovian cue-cocaine memory was evidently spared by the MK-801 + VR5 reactivation reconsolidation manipulation, as the cocaine cue retained its ability to modulate cocaine-seeking. This implies that pavlovian CS associations were not destabilized by the VR5 reactivation session and demonstrates that instrumental and pavlovian memories that share the same rewarding outcome can be selectively destabilized. This complements previous demonstrations that reactivation sessions promoting the destabilization of pavlovian CS-cocaine associations do not affect the underlying lever pressing, suggesting intact instrumental memory and hence selectivity to pavlovian memory destabilization (Lee et al., 2006; Milton et al., 2008a). While these observations reinforce our conclusion that instrumental memory was disrupted in the present study, they also raise an important issue for clinical translation in that the destabilization and/or reconsolidation of both pavlovian and instrumental memories have not yet been shown to be possible within a single reactivation session. An important caveat, however, to these conclusions is the apparent discrepancy in the training history of the CS relapse rats in our Experiment 2 (*μ* = 216 total cocaine infusions), compared to the rats in the other experiments (*μ* = 148–164 across experiments). It is possible that the greater strength of the conditioned instrumental cocaine memory resulting from the increased number of cocaine infusions may have provided a boundary condition on instrumental memory destabilization under the present reactivation parameters. If this were the case, the apparent recovery with CS presentation would in fact, reflect a lack of underlying instrumental impairment, leaving open the possibility that disrupted instrumental cocaine memory reconsolidation is not recovered by response-contingent CS presentation. Such a conclusion is, however, weakened by our observations in experiment 4, in which the groups treated with vehicle, rather than yohimbine, also show no evidence for an effect of MK-801 in a test of cue-induced relapse despite the markedly reduced cocaine intake history.

It is particularly notable that our reactivation protocol did not destabilize pavlovian memory, as previous studies have successfully destabilized cocaine memory with simple brief CS exposure (Lee et al., 2006) and our VR5 reactivation also included CS presentation. Operationally it appears that which memory trace is destabilized depends upon the functional features of the reactivation (i.e., what new information is presented). Changing the response-reward contingency destabilizes instrumental memory (Exton-McGuinness et al., 2014) while altering cue-reward contingency causes pavlovian memories to undergo reconsolidation (Lee et al., 2006; Milton et al., 2008b). However, it remains unclear how destabilization of pavlovian and instrumental memories interact with each other.

Returning to the lack of stress-induced relapse, it is worth noting that yohimbine, and stress more generally, appear to have strong effects upon CS-mediated forms of reinstatement (Feltenstein and See, 2006; Banna et al., 2010). Our results do not show clearly whether or not yohimbine, while ineffective in recovering spontaneous cocaine-seeking, is able to act synergistically to enhance cue-induced relapse. In our Yohimbine+CS experiment, yohimbine did not elevate cue-induced cocaine-seeking in MK-801-treated rats, even though it did increase responding in saline-treated controls. This suggests an inability of stress to potentiate cue-induced cocaine-seeking following instrumental memory reconsolidation impairment. In contrast, there was little statistical evidence for an effect of MK-801, compared to saline, on yohimbine-stimulated cue-induced cocaine-seeking at test, indicating that yohimbine might be able to recover behavior to near-normal levels. Inspection of the numerical results, however, suggests that the MK-801-treated rats did respond at lower levels than saline controls. Moreover, the failure of yohimbine to elevate seeking in MK-801-treated rats was certainly not due to any ceiling effect. Therefore, our results are most consistent with the conclusion that the previous failure of yohimbine to recover impaired cocaine-seeking is not simply due to the absence of the CS at test.

In contrast to yohimbine, and similarly to cue-induced relapse, cocaine priming was effective in recovering cocaine-seeking at test. It should be noted that the cocaine-induced relapse test was conducted in the absence of explicit cues, a setting previously demonstrated to attenuate amphetamine-primed reinstatement of amphetamine seeking (Stretch et al., 1971); nevertheless, there was evidence that cocaine priming did quantitatively elevate responding, at least with visual inspection of the MK-801 groups.

The present pattern of results, with protection afforded only to stress-induced relapse, informs the important question of how cocaine priming and response-contingent CS presentation restored cocaine-seeking, given the background of an instrumental memory impairment. There are several potential accounts for such recovery. A first explanation focusses on the capacity of cues and cocaine to induce craving for the cocaine outcome, which can drive instrumental responding (Schmidt et al., 2005; Shaham and Hope, 2005). However, such an account relies upon responding being only partially disrupted, as was seemingly the case in our results. Why recovery is statistically complete, rather than demonstrating enhancement of impaired responding but to lower levels than control, is unclear but may simply reflect ceiling effects, rather than a true complete recovery. Nevertheless, the lack of recovery following yohimbine treatment is not easily explained within a craving framework, as stress has similarly been argued to precipitate relapse *via* induction of craving (Sinha et al., 1999) and yohimbine can similarly induce objective and subjective stress, and accompanying drug-craving (Stine et al., 2002). Perhaps then, a focus on drug sensitization may be more relevant, given first the difficulty in applying the concept of craving to rodent behavior, and more importantly the dissociation between CS and cocaine effects on mesocorticolimbic dopamine, as opposed to yohimbine effects on CRF release (Stewart, 2000; Shalev et al., 2002, 2010). Moreover, while drug self-administration can sensitize stress response systems (Sarnyai et al., 2001), resulting in enhanced stress-induced responding (Ahmed et al., 2000; Shalev et al., 2001), the latter are typically observed with longer access to cocaine or longer training histories than those used in the present study.

A second explanation for the recovery in cue- and cocaine-induced relapse tests appeals to the debate surrounding the nature of reconsolidation impairments. While traditionally considered to reflect storage impairments (Lee, 2009), it has also been argued that performance deficits result from state-dependent learning effects (Millin et al., 2001) or, more recently, integration of new information (Gisquet-Verrier et al., 2015). The latter hypotheses emphasize observations of recovery from amnesia. Cocaine-primed and cue-induced reinstatement are predicated, at least in part, on the capacity of cocaine and cues to remind and reinstate the extinguished and inhibited instrumental memories (Shalev et al., 2002). Yohimbine-induced stress would not necessarily be expected to act as a reminder for the disrupted instrumental memory, and so the lack of recovery in the present results might be taken as support for retrieval impairment accounts of instrumental memory reconsolidation deficits. However, the different nature of stress-induced reinstatement might also point towards reconsolidation being a storage deficit in the current study. Stress has been argued, albeit typically when discussing footshock stress, to interfere with behavioral inhibition, thereby releasing instrumental responding from inhibition and leading to relapse (Shaham et al., 2000). While this can explain stress-induced reinstatement of instrumental responding that is under inhibition, either following extinction or other forms of retrieval inhibition, it would mean that stress would not be expected to recover a genuine impairment in instrumental memory storage.

Notably instrumental behaviors can be mediated *via* either a “goal-directed” Action–Outcome (A–O) memory, or a Stimulus–Response (S–R) “habit” (Dickinson, 1985). Previous studies of reconsolidation have not demonstrated the disruption of more than one association following a single reactivation and drug treatment. Therefore, it is perhaps likely that only one of the A–O and S–R memories was destabilized and its reconsolidation impaired. Given our conclusion in sucrose seeking that reactivation and MK-801 impaired the S–R association (Exton-McGuinness et al., 2014), we might assume that the A–O memory remained intact. This adds a further level of complexity to the explanation for recovery of responding, as it might not reflect recovery of the impaired S–R association, but rather activation/enhancement of the preserved A–O memory, especially as goal-directed responding appears to be actively inhibited in the course of the development of S–R habits (Coutureau and Killcross, 2003). Such an activation might be achieved *via* a reminder process, for which exposure to the CS and cocaine would again be expected to be effective. In contrast, there is little evidence that stress can activate instrumental responding; for example, single behavioral stress prior to a test for pavlovian-instrumental transfer test did not increase baseline instrumental responding (Pielock et al., 2013).

In summary, reconsolidation-disruption of instrumental memory supporting cocaine-seeking can protect against stress-induced relapse, as well as diminish spontaneous rates of responding. However, little protection is afforded to cue-induced and cocaine-induced relapse. This leaves open the possibility that combined targeting of both instrumental and pavlovian memories might provide greater resistance to relapse. Moreover, a memory reactivation through non-contingent administration of cocaine can protect against cocaine-induced relapse (Luo et al., 2015). Interestingly, if it is the case that the targeting of instrumental memory reconsolidation disrupts selectively the S–R “habit” associations that are believed to be key in driving addiction (Milton and Everitt, 2012), this may leave behavior under adaptive goal-directed control. This highlights the importance of translating the current findings into experimental settings that evaluate addition-like behavior (e.g., Belin et al., 2008), as simple cocaine-seeking does not necessarily afford insight into whether the measured behavior is genuinely maladaptive.

## Data Availability Statement

The datasets generated for this study are available on request to the corresponding author.

## Ethics Statement

The animal study was reviewed and approved by University of Birmingham Animal Welfare and Ethical Review Board and carried out in accordance with the UK Animals (Scientific Procedures) Act 1986, Amendment Regulations 2012 (PPL P8B15DC34 and PPL P3B19B9D2).

## Author Contributions

M-EM designed the experiments, conducted the research and wrote the article. JL designed the experiments and wrote the article. MD and CF conducted the research.

## Conflict of Interest

The authors declare that the research was conducted in the absence of any commercial or financial relationships that could be construed as a potential conflict of interest.

## Abbreviations

CS: conditioned stimulus
NMDAR: N-methyl–D-aspartate receptor
VR5: variable ratio 5.
